# A Comparative Study of Feature Selection Approaches for Human Activity Recognition Using Multimodal Sensory Data

**DOI:** 10.3390/s21072368

**Published:** 2021-03-29

**Authors:** Fatima Amjad, Muhammad Hassan Khan, Muhammad Adeel Nisar, Muhammad Shahid Farid, Marcin Grzegorzek

**Affiliations:** 1 Punjab University College of Information Technology, University of the Punjab, Lahore 54000, Pakistan; MSCSF17M525@pucit.edu.pk (F.A.); muhammad.nisar@student.uni-luebeck.de (M.A.N.); shahid@pucit.edu.pk (M.S.F.); 2 Institute of Medical Informatics, University of Lübeck, Ratzeburger Allee 160, 23538 Lübeck, Germany; grzegorzek@imi.uni-luebeck.de

**Keywords:** human activity recognition, subspace pooling, atomic actions, composite activities

## Abstract

Human activity recognition (HAR) aims to recognize the actions of the human body through a series of observations and environmental conditions. The analysis of human activities has drawn the attention of the research community in the last two decades due to its widespread applications, diverse nature of activities, and recording infrastructure. Lately, one of the most challenging applications in this framework is to recognize the human body actions using unobtrusive wearable motion sensors. Since the human activities of daily life (e.g., cooking, eating) comprises several repetitive and circumstantial short sequences of actions (e.g., moving arm), it is quite difficult to directly use the sensory data for recognition because the multiple sequences of the same activity data may have large diversity. However, a similarity can be observed in the temporal occurrence of the atomic actions. Therefore, this paper presents a two-level hierarchical method to recognize human activities using a set of wearable sensors. In the first step, the atomic activities are detected from the original sensory data, and their recognition scores are obtained. Secondly, the composite activities are recognized using the scores of atomic actions. We propose two different methods of feature extraction from atomic scores to recognize the composite activities, and they include handcrafted features and the features obtained using the subspace pooling technique. The proposed method is evaluated on the large publicly available CogAge dataset, which contains the instances of both atomic and composite activities. The data is recorded using three unobtrusive wearable devices: smartphone, smartwatch, and smart glasses. We also investigated the performance evaluation of different classification algorithms to recognize the composite activities. The proposed method achieved 79% and 62.8% average recognition accuracies using the handcrafted features and the features obtained using subspace pooling technique, respectively. The recognition results of the proposed technique and their comparison with the existing state-of-the-art techniques confirm its effectiveness.

## 1. Introduction

**Figure 1:**
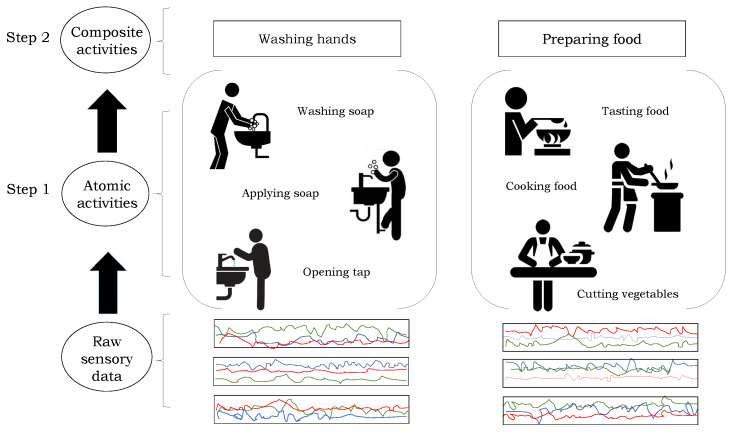
Hierarchical model to recognize human activities. In the first step, the atomic activities are detected from the original sensory data, and their recognition scores are obtained, which are used to recognize the composite activities in the second step.

Human activity recognition (HAR) aims at verifying the individual’s activity of daily livings (ADLs) from the data captured using various multimodal modalities, e.g., time-series data from motion sensors, color and/or depth information from video cameras. Lately, HAR-based systems have gained the attention of the research community due to their use in several applications, such as healthcare monitoring applications, surveillance systems, gaming applications, and anti-terrorist and anti-crime securities. The availability of low cost yet highly accurate motion sensors in mobile gadgets and wearable sensors, e.g., smartwatches, has also played a vital role in the dramatic development of these applications. The main objective of such a system is to automatically detect and recognize the human daily life activities from the captured data by creating a predictive model that allows the classification of an individual’s behavior [1]. ADL refers to such tasks or activities that undertake by people in their daily life [2]. An ADL is usually a long term activity that consists of a sequence of small actions as depicted in Figure 1. We can describe the long term activities as composite activities, e.g., cooking, playing, etc., whereas the small sequence of actions are known as atomic activities, such as raising an arm or a leg [3].

**Figure 2:**
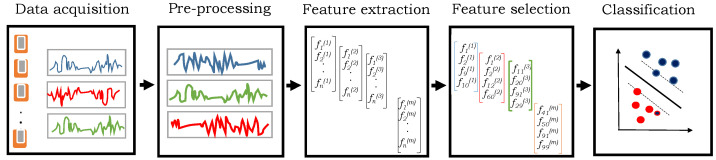
Different steps to recognize human activities from raw sensory data.

HAR systems usually follow a standard set of actions in a sequence which includes data capturing, pre-processing, feature extraction, the selection of most discriminant features, and their classification into the respective classes (i.e., activities) [4] as depicted in Figure 2. The first step is the data collection using the setup of wearable sensors, force plates, cameras, etc. The selection of such a sensor is very much dependent on the nature of ADL to be recognized. In many cases, it is inappropriate to use raw sensor data directly for activity recognition because it may contain noise or irrelevant components. Therefore, data pre-processing techniques, such as normalization, recovery of missing data, unit conversion, etc., are applied in the second step to make the raw data suitable for further analysis. Third, the high-level features are extracted on this pre-processed data based on the expert’s knowledge in the considered application domain. Usually, several features are extracted and the most important of them are selected such that they retain the maximum possible discriminatory information. This process is known as feature selection which not only reduces the dimensions but also reduces the need for storage and computational cost. Finally, the features are recognized into respective activities using the classifier which provides the separation between the features of different activities in the feature space. It is worth mentioning that the performance of the HAR system is very much dependent on every step of the sequence.

Numerous researchers have proposed handcrafted features on visual [5] and time series data [6] to detect and recognize human activities. However, there is no guarantee that the engineered handcrafted features work well for all the scenarios. Many factors, such as the nature of input data and prior knowledge, in the application domain, play an important role to extract the optimal features. Therefore, the researchers are continuously trying to explore more systematic ways to get the optimal features. Lately, deep learning-based HAR systems, e.g., References [7,8], have also explored to extract the features using raw data of wearable sensors automatically. These systems exploited Artificial Neural Networks (ANNs), which consist of multiple artificial neurons, arranged and connected in several layers such that the output of the first layer is forwarded as an input to the second layer, and so forth. That is, each next layer is capable to encode the low-level descriptors of the previous subsequent layer. Hence, the last layer of the deep ANN provides highly abstract features from the input data. Though the deep learning-based techniques have demonstrated excellent results on various benchmark datasets, it is, however, quite difficult to rigorously assess the performance of feature learning methods. Despite their good performance, they need a large amount of training data to tune the hyper-parameters. The lack of knowledge in the implementation of optimal features [4,9,10], the selection of the relevant features that represent the ongoing activity [11,12], and the parameters in the classification techniques [13,14] make the whole process much complicated.

This paper presents a novel two-level hierarchical method to recognize human activities using a set of wearable sensors. Since the human ADL consists of several repetitive short series of actions, it is quite difficult to directly use the sensory data for activity recognition because the two or more sequences of the same activity data may have large diversity. However, a similarity can be observed in the temporal occurrence of the atomic actions. Therefore, the objective of this research is to analyze the recognition score of every atomic action to represents a composite activity. To solve this problem, we propose a two-level hierarchical model which detects atomic activities at the first level using raw sensory data obtained from multiple wearable sensors, and later the composite activities are identified using the recognition score of atomic activities at the second level. In particular, the atomic activities are detected from the original sensory data and their recognition scores are obtained. Later, the features are extracted from these atomic scores to recognize the composite activities. The contribution of this paper is two-fold. First, we propose two different methods for feature extraction using atomic scores: Handcrafted features, and the features obtained using subspace pooling technique. Second, several experiments are performed to analyze the impact of different hyper-parameters during the computation of feature descriptors, the selection of optimal features that represent the composite activities, and the evaluation of different classification algorithms. We used the CogAge dataset [15] to evaluate the performance of our proposed algorithm which contains the data of 7 composite activities performed by 6 different subjects in different time intervals using three wearable devices: smartphone, smartwatch, and smart glasses. Each of the composite activities can be represented using the combination of 61 atomic activities. We considered each atomic activity as a feature hence, a feature vector of 61 dimension is used to represent the composite activity. The recognition results are compared with existing state-of-the-art techniques. The recognition results of the proposed technique and their comparison with the existing state-of-the-art techniques confirm its effectiveness.

The rest of this paper is organized as follows: a brief review of literature on human activity recognition is presented in Section 2. The overview of the proposed method is described in Section 3. The proposed activity recognition algorithm is presented in Section 4. The experimental evaluation through different qualitative and quantitative tools is carried out in Section 5. The conclusion is drawn in Section 6.

## 2. Background and Literature Review

Human activity recognition has gained the interest of the research community in the last two decades due to its several applications in surveillance systems, rehabilitation places, gaming, and others. This section summarizes the most existing work in the field of human activity recognition using sensor-based time-series data.

A composite activity comprises several atomic actions. Numerous existing techniques focused on identifying the simple and basic human actions, whereas recognizing the composite activities remains an active problem. The applications in daily life require the identification of high-level human activities which are further composed of smaller atomic activities [16]. This paper summarizes the existing piece of work on hierarchical activity recognition techniques to encode the temporal patterns of composite activities. The techniques proposed in References [17,18] have concluded that the hierarchical recognition models are effective to recognize human activities. The authors in Reference [19] presented a method to recognize the cooking-related activities in visual data using pose-based, hand-centric and holistic approaches. In the first step, the hand positions and their movements are detected and then the shape of the knife and vegetable is determined. Lastly, the composite activities are recognized using fine-grained activities. The technique in Reference [20] proposed a hierarchical discriminative model to analyze the composite activities. They employed a predefined sequence of atomic actions and reported improvements in recognition accuracy when the composite activities are recognized using a hierarchical approach. In Reference [21], a hierarchical model is proposed to recognize the human activities using accelerometer sensor data of smartphone. The technique proposed in Reference [22] employed a two-level classification method to recognize 21 different composite activities using a set of wearable devices on multiple positions of the human body. The authors in Reference [23] presented an approach to detect the primitive actions in the recorded data of composite activities which were used to recognize the ADLs using the limited amount of training data. The technique proposed in Reference [24] employed a Deep Belief Network to construct the mid-level features which were used to recognize the composite activities. In comparison with the aforementioned techniques, we propose a more generic and hierarchical technique to recognize the composite activities using the score of underlying atomic activities. In the first step, the score of atomic activities is computed directly from the input data. Later, the scores of atomic activities are used to recognize the composite activities. The atomic activities are defined manually to make our hierarchical approach more general. Though this paper mainly emphasizes the recognition of 7 composite activities (available in the selected dataset), we, however, believe that many other composite activities can also be recognized by including more atomic activities describing the variational movements of the human body, which reflects the generality of the proposed technique.

In Reference [25], the authors reviewed the existing literature on human activities in the aspect that how they are being used in different applications. They concluded that activity recognition in visual data is not much effective due to the problems of clutter background, partial obstruction, changing of scale, illumination, etc. Lara et al. [6] surveyed the state-of-the-art techniques to recognize human activity using wearable sensors. They explained that obtaining the appropriate information on human actions and behaviors is very important for their recognition, and it can be efficiently achieved using sensory data. The authors in References [26,27,28,29] have also assessed the use of wearable sensors for human activity recognition. Shirahama et al. [30] proposed that the current spread of mobile devices with multiple sensors may help in the recognition and monitoring of ADL. They employed a codebook-based approach to get the high-level representation of the input data which was obtained from different sensors of the smartphone. The technique proposed in Reference [31] recognizes the human activities using the sensory data which is obtained from 2-axes of the smartphone accelerometer sensor. This research also concluded the effectiveness and contribution of each axis of the accelerometer in the recognition of human activities. In comparison with the aforementioned techniques, the proposed technique uses multimodal sensory data which is obtained from three unobtrusive wearable devices. We show that the fusion of multimodal sensory data provides higher accuracy to recognize the ADL.

In machine learning algorithms, the selection of optimal features plays a vital role to obtain excellent recognition results. Furthermore, in the case of high-dimensional data, the reduction of dimensions may not only help to improve the recognition accuracies but also to reduce the memory requirement and computational cost [32]. The dimensionality reduction usually can be achieved using two techniques: feature selection and feature extraction [33,34]. The authors in Reference [35] proposed a hybrid approach by employing both feature selection and feature extraction techniques for dimension reduction. Lately, a few authors, e.g., References [36,37], proposed feature extraction methods using the subspace pooling technique. The technique proposed in Reference [36] employed singular value decomposition (SVD) for subspace pooling to obtain the optimal set of features from high dimensional data. The authors in Reference [38] extracted a set of features using SVD and the principal singular vectors to encode the feature representation of input data. Zhang et al. [37] also employed SVD for subspace pooling technique in their work. Guyon et al. [39] have discussed multiple methods of feature selection in their research and concluded that clustering and matrix factorization performed best when the dimensions became very large. Similarly, the techniques proposed in References [40,41,42] have also employed SVD for optimal features selection. Song et al. [43] exploits principal component analysis (PCA) to select the most prominent and important features. They concluded that the recognition accuracy remained the same even after reducing the dimensions by selecting a few components. The authors in References [44,45] have also used PCA for optimal feature selection.

The researchers have also assessed the performance of different classification algorithms to recognize the ADL. For example, the authors in Reference [46] employed several machine learning algorithms on multivariate data to recognize human activities. They assessed the performance of Random Forest (RF), kNN, Neural Network, Logistic Regression, Stochastic Gradient Descent, and Naïve Bayes and concluded that Neural Network and logistics regression techniques provide better recognition results. In Reference [14], the authors assessed the different kernels of SVM to recognize the ADL which were recorded using Inertial sensors. The authors in Reference [13] concluded that the selection of kernel function in SVM along with the optimal values of hyperparameters plays a critical role concerning the data. The authors in Reference [47] have also used SVM as a classification tool in their research. Yazdansepas et al. [48] proposed a method to recognize the human activities of 77 subjects. They assessed the performance of different classification algorithms and concluded that the random forest algorithm provides the best results. The hidden Markov model has been widely used for activity recognition [49,50,51,52]. It is a sequential probabilistic model where a particular discrete random variable describes the state of the process. The technique proposed in Reference [22] employed Conditional Random Fields (CRFs) to encode the sequential characteristics of composite activity. Deep learning-based techniques, e.g., References [7,8], have also been employed to recognize human activities. Despite their good performance, they need a large amount of training data to tune the hyper-parameters [53]. The ensemble classifier (i.e., combined predictions of several models) have been also employed to recognize the ADL. For example, the authors in Reference [54] presented that ensemble classifiers gave more accurate results than any other single classifier. Mishra et al. [55] explained that the increase in variety and size of data affect the performance of a classifier. They concluded that estimations of more than one classifier (i.e., ensemble classification) are required to improve the performance. Similarly, the authors in Reference [56] employed an ensemble classifier using a voting technique in the classification of patterns. They formed a few sets of basic classifiers which were trained on different parameters. They combined their predictions by using a weighted voting technique to get a final prediction. Their research showed that ensemble classifier is quite a promising method, and it might get popular in other science-related fields. There were many techniques for ensemble classification but the voting-based technique is an efficient one.

In comparison with existing techniques, this paper presents a generic activity recognition method using the subspace pooling technique on the scores of atomic activities which were computed from the original sensory data. In particular, two different types of features are computed from the atomic scores, and their performance is assessed using four different classifiers with different parameters. The performance evaluation and its comparison with existing state-of-the-art techniques confirm the effectiveness of the proposed method.

## 3. Overview of the Proposed Method

The analysis of human activities has gained the attention of the research community due to its use in several daily life applications. Typically these activities are recorded using multiple electronic devices. In this paper, we used the CogAge dataset [15] which is collected by using three unobtrusive wearable devices: smartphone, smartwatch, and smart glasses. In particular, the LG G5 smartphone was placed in the proband’s front left pocket of the jeans. The mobile device is used to capture body movement. Second, the Huawei smartwatch was placed on a subject’s left arm to record the movements of the hand. Third, the JINS MEME glasses are worn by the subject to get the head movement. Since the human activity of daily life consists of several repetitive and concurrent short sequences of actions, they cannot be directly estimated from the sensory data because the multiple sequences of the same activity data may have large diversity. However, a similarity can be observed in the temporal occurrence of the atomic actions. A two-level hierarchical method is presented to recognize human activities. First, the scores of the atomic activities are obtained. Secondly, two different types of features are extracted using the score of atomic actions. The proposed features are evaluated using different classification algorithms. The recognition results of the proposed technique and their comparison with the existing state-of-the-art techniques are presented.

## 4. Proposed Method

This section presents the proposed two-level hierarchical model for composite activity recognition. In the first step, we employed a codebook-based approach to get the recognition scores of atomic activities. Second, different features are extracted on these scores to recognize the composite activities. The description of each of the two steps is outlined in the following subsections.

### 4.1. Recognition of Atomic Activities

We recall that an ADL is usually a long term activity (i.e., composite activity) and consists of a sequence of small actions (i.e., atomic activities). The recognition scores of the atomic activities are computed as described in our earlier paper (References [4,15]) and used as input data to our framework to describe the composite activity. The complete scenario is described in the following to assist the reader.

**Figure 3:**
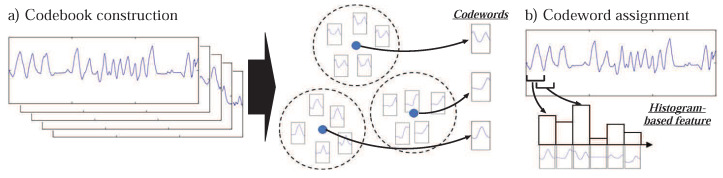
The depiction of codebook-based feature extraction process: (**a**) Codebook construction by grouping the similar subsequences using k-means clustering algorithm. The center of each group is set as “codeword”. (**b**) Features are computed on each of the subsequences by assigning them to the most similar codeword [4].

To recognize the atomic activities, the multi-dimensional data is recorded using the aforementioned sensors. The recognition process consists of two main phases: “Feature extraction” and “training/test of model”. From each of the sensors, the features are extracted using a codebook-based approach and they are fused into a single high-dimensional feature. The codebook-based approaches compute a histogram-type feature depicting the frequencies of characteristic subsequences, called codewords, in a sequence [57]. They first construct a codebook by grouping the similar subsequences using any clustering algorithm (we used K-means), whereas the subsequences are collected by following a sliding window approach. The center of each group is set as “codeword”. Later in the second step, the features are computed on other subsequences by assigning them to the most similar codeword. Therefore, the resulting feature represents the repetition of each codeword in the sequence (i.e., histogram) as depicted in Figure 3. The codebook-based features are extracted from the sequences of each sensor, and they are fused using the strategy of early fusion [10]. It concatenates all the features into a high-dimensional feature vector. That is, each atomic activity is represented as a single high-dimensional feature vector which is used to train the classification algorithm. After training, the unknown sequences (i.e., test sequences) are passed to the trained model and their atomic scores are obtained which represent the probability of an atomic activity in the test instance. Since the final feature vector is high-dimensional (after fusion), we used Support Vector Machine (SVM) [58] with a one-versus-all approach due to its effectiveness for high-dimensional data [59]. The model is trained to produce a scoring value between 0 and 1 as an output that represents the score of the atomic activity. That is, the large score means the more likely the example includes the atomic activity. This score is computed based on the distance between the subsequence example and the classification boundary in the high-dimensional space. We trained *n* number of SVMs to get the *n* atomic scores for a testing example. In the online setting, the *n* number of SVMs calculate the atomic scores for the sequences recorded from different sensors in a certain time interval. Since this paper mainly emphasizes the recognition of composite activities, we refer to the reader to review the details of feature computation of atomic activities in our earlier paper (References [4,15]). The details can also be found on the web page of our earlier paper (Reference [60]).

### 4.2. Recognition of Composite Activities

The objective of the proposed research is to recognize the composite activities using the scores of *M* atomic activities which were computed by following the method described in Section 4.1. Suppose that, for *N* composite activities, we have *K* instances, and the *k*th instance of a composite activity $X^{\left(k\right)}\left(1\leq k\leq K\right)$ has a length of $T_{k}$ time points. Mathematically, it can be described as,

(1) $$X^{\left(k\right)}=\left({x_{1}}^{\left(k\right)},{x_{2}}^{\left(k\right)},{x_{3}}^{\left(k\right)},\ldots ..{x_{T}}^{\left(k\right)}\right),$$

where each ${x_{i}}^{\left(k\right)}\in R^{M}$ represents a vector of *M* atomic scores at given time $i(1\leq i\leq T_{k})$ for $X^{\left(k\right)}$. The term $x^{\left(k\right)}$ can be described as,

(2) $${x_{i}}^{\left(k\right)}=\left({x_{i1}}^{\left(k\right)},{x_{i2}}^{\left(k\right)},{x_{i3}}^{\left(k\right)},\ldots .,{x_{ij}}^{\left(k\right)},\ldots ..,{x_{iM}}^{\left(k\right)}\right),$$

where ${x_{ij}}^{\left(k\right)}$ has the value in the range between 0 and 1 and describes the atomic score of *j*th atomic activity (1≤j≤M), computed at time *i* for $X^{\left(k\right)}$. Several high-level features are extracted from these atomic scores (computed in Section 4.1) to encode the composite activities. In particular, we employed two different feature extraction techniques: handcrafted features and subspace pooling-based features. The detail of each of the technique is described in the subsequent sections.

#### 4.2.1. Handcrafted Features

Handcrafted feature extraction techniques are simple to implement and lower in computational complexity. They can be computed on time series data either using simple statistical operations (e.g., finding maximum, minimum, average, standard deviation, etc.), or more detailed components, such as frequency domain-based features, which are related to the Fourier transform of the signals [61]. We computed 18 handcrafted features on the data of atomic scores in which 15 features are computed using statistical formulation and the rest are based on frequency domain. The list of the computed features is summarized in Table 1, and their description is outlined in the following. These statistical values were computed to every feature dimension separately.

- Maximum: Let *X* is the feature vector. The Max(X) function finds and returns the largest feature value $x_{i}\in X$.
- Minimum: The Min(X) function finds and returns the smallest feature value $x_{i}\in X$.
- Average: For *N* number of feature values, the average returns the center value of feature vector *X*. That is,
  (3) $$Average\left(X\right)=\mu =\frac{\sum_{i=1}^{n}x_{i}}{N}.$$
- Standard Deviation: It describes the amount of disparity in feature vector $X=\{x_{1},x_{2}\ldots x_{N}\}$ and can be computed using the following formulation:
  (4) $$Stdev\left(X\right)=\sigma =\sqrt{\frac{1}{N}\sum_{i=1}^{N}{\left(x_{i}-\mu \right)}^{2}}.$$
- Zero Crossing: It is used to estimate the difference between a rapid and slow movement of activity [61] and can be calculated by estimating how often the signal value crosses zero in either direction.
- Percentiles: Percentile defines a number where a certain percentage of scores fall below that number. That is, the *p*th percentile is a value such that, at most, (100×p)% of the measurements fall below than this value, and 100×(1−p)% of the measurements fall above this value. For instance, the 25th percentile means that this value is bigger than 25 values and smaller than 75 feature values. The 25th percentile is also called the first quartile. The 50th percentile is generally the median, and the 75th percentile is also called the third quartile.
- Interquartile Range: The difference between the third and first quartiles is known as the interquartile range.
- Skewness: It calculates the asymmetry of the probability distribution of data about its mean and can be calculated as:
  (5) $$Sk=\frac{1}{N\sigma^{3}}\sum_{i=1}^{n}{\left(x_{i}-\mu \right)}^{3}.$$
- Kurtosis: It is the measure that how heavily the tails of distribution differ from the tails of a normal distribution. The higher value of kurtosis corresponds to the greater extremity of deviations which refer to outliers [62]. Mathematically, it can be computed as:
  (6) $$Kr=\frac{1}{N\sigma^{4}}\sum_{i=1}^{n}{\left(x_{i}-\mu \right)}^{4}.$$
- Auto-correlation: It measures the degree of similarity between a given time series data and a lagged version of itself over the successive time intervals. That is, it depicts the notch of similarity between a current feature value and its earlier values [63], and it can be computed as:
  (7) $$r_{k}=\frac{\sum_{i=1}^{N-k}\left(x_{i}-\mu \right)\left(x_{i+k}-\mu \right)}{\sum_{i=1}^{N}{\left(x_{i}-\mu \right)}^{2}}.$$
- Order mean values: They are computed from the arranged set (increasing order) of values. That is, the first-order mean is the simple smallest sample value $x_{1}$ in an arranged feature set *X*, the second-order defines the second smallest value $x_{2}$, and so forth [64].
- Norm values: They are used to estimate the distance of a feature vector from its origin. We used two different measures: $L_{1}$-norm (also known as Manhattan distance) and $L_{2}$-norm (also known as Euclidean norm) [65].
- Spectral energy: We recall that several sensors are used in the recorded data to analyze human activities, and they can be considered as a function whose amplitude is changing over time. We used Fourier transformation to transform the time-based signal to its frequency spectrum, and spectral energy formulation is employed to calculate the signal energy distribution across the frequency. It measures the sum of squared amplitude of frequency content ϝ(n). That is,
  (8) $$S_{E}=\sum_{i=1}^{N}ϝ{\left(n\right)}^{2}.$$
  It can also be computed using normalized frequency spectra. That is,
  (9) $$\hat{ϝ}\left(n\right)=\frac{ϝ(n)}{\sum_{i=1}^{N}ϝ\left(n\right)}.$$
  After the normalization process, the Equation (8) can be described as,
  (10) $$NS_{E}=\sum_{i=1}^{N}\hat{ϝ}{\left(n\right)}^{2}.$$
- Spectral entropy: It is based on the concept of the Shannon entropy and is used to measure the spectral of the signal distribution in terms of frequency. The mathematical formulation of spectral entropy can be described as:
  (11) $$S_{EN}=-\sum_{i=1}^{N}\hat{ϝ}\left(n\right)\times \log\hat{ϝ}\left(n\right).$$

#### 4.2.2. Subspace Pooling

The subspace pooling-based technique aims to model/project the original complex input data into new dimensions such that the basic structure of data can be analyzed easily and accurately [66]. Thus, rather than working on the original input space, further processing will be applied on a more robust representation of data in subspace [67]. Such techniques, e.g., Reference [36], have proven to be effective in extracting the temporal features for activity recognition. Therefore, we also applied subspace pooling techniques on our recorded data using singular value decomposition (SVD) [68] and principal component analysis (PCA) [69] techniques. SVD is the decomposition of the complex matrix into three simple matrices, whereas PCA defines the orthogonal projection of original data to the new dimensions. They maximize the variance of the projected data [70] such that the interesting properties of the original data are described. In particular, the objective of these techniques is to reduce the complexity of data by converting the original data to less complex subspace data with the help of eigenvectors and eigenvalues. They are the well-known techniques of numerical linear algebra system and have been extensively used to reduce the complexities in original high dimensional data [71].

**Figure 4:**
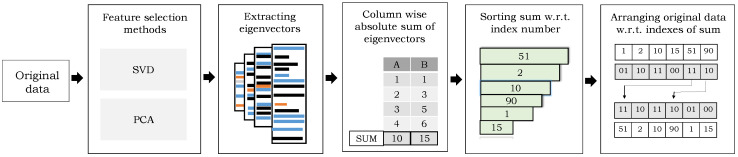
An overview of the feature selection process using subspace pooling techniques.

Since the real world, data may contain redundant, noisy, and irrelevant features which make the learning process complex; the removal of such features will not only reduce the complexity in the model learning but also reduce the need for storage and computational cost [32]. Another simple yet powerful use of the SVD and PCA is that they can also be used in the selection of optimal features by discarding the irrelevant information [72]. Therefore, we also employed them in the feature selection process to select the most influential features of original data that are having a greater impact on the recognition of composite activity. The entire process is explained in the following and depicted in Figure 4.

- Step 1: The aforementioned subspace pooling techniques are applied on original multidimensional data (i.e., the recognition score of the atomic activities).
- Step 2: Eigenvectors of n×n dimensions are extracted using SVD and PCA, where n=18×61=1098.
- Step 3: The absolute sum of every single column of eigenvectors with dimension n×1 is computed.
- Step 4: The sorting algorithm is applied on the sum of eigenvectors with respect to the index to assess the importance of every feature (obtained after subspace pooling technique). The feature with the highest sum is considered the most important feature.
- Step 5: The original data (i.e., atomic scores) is arranged with respect to the sorted sum.

### 4.3. Classification

We evaluated the performance of our proposed features using different classification techniques. A short description of each of the technique is described in the following:

- Support Vector Machine (SVM) [73] is a simple and well-known supervised machine learning algorithm to solve the problem of classification. SVM first maps the training instances into high dimensional space and then extracts a decision boundary (e.g., hyperplane) between the instances of different classes based on the principle of maximizing the margin (i.e., the distance between the hyperplane and the nearest data point in each class is maximized). The training instances which are very close to the class boundary are known as support vectors. The training process aims to find such a hyperplane that should be in the middle of positive and negative instances, and the distance of hyperplane with the nearest positive and negative instances should be maximized [3,74]. We used simple LibLinear SVM [73], and the optimal value or hyperparameter/regularization parameter *C* is selected empirically within the range of ($2^{-6}$– $2^{6}$).
- RF [75] is an ensemble learning algorithm that uses multiple individual learners and fuses the results. In particular, it comprises the collection of decision trees with a random subset of data, and the outputs of all the decision trees are then combined to create the final decision (i.e., recognition). The regularization parameter *n* is selected empirically in the range of 600–2000 with the increment size of 200.
- The Hidden Markov Model (HMM) [76] has also been employed as a classifier to recognize the human actions [77,78] using a few unobservable (i.e., hidden) states, and sequences whose behavior depends on the hidden states [79]. The model is usually constructed using a set of states with a stochastic matrix storing the transition information between each state. The elements of such a matrix hold probabilities of states over time known as transition probabilities. Every state in HMM is associated with a probability density function (PDF) which helps in determining that every state emits a sequence over time known as observation sequence. The main objective of HMM is to learn the behavior of hidden states based on observable sequences [80].
- The idea of ensemble classifier has also attracted an increasing amount of attention from the research community due to its effectiveness in the field of pattern recognition [81]. Ensemble classifier aims that the recognition technique not to depend on a decision of a single classification model, rather the final decision is based on the combination of several individual classifier [82]. It can be built by training several individual classifiers (also known as base learners) and combining their results/estimations by voting (either weighted or unweighted). That is, the estimations of all classification algorithms are combined so that votes with the highest number (i.e., maximum occurrences) are considered as the final ensemble prediction [83]. It is quite a known fact that ensemble classifiers give more accurate results than the other individual classifiers (base learners) from which they have been built.

## 5. Experiments and Results

**Figure 5:**
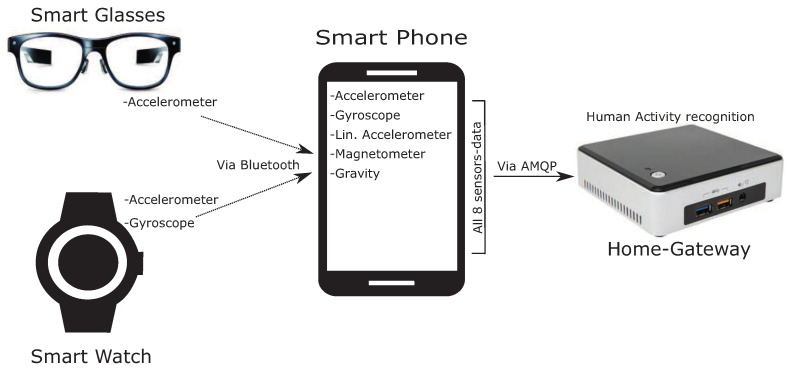
Activity recording setup using three wearable devices. The movement data of smart glasses and watch is initially sent to smartphone via Bluetooth connection and later all the recorded data sent to a home-gateway using Wi-Fi connection [15].

We used the CogAge dataset [15], which contains both atomic and composite activities. The data is recorded using three unobtrusive wearable devices: LG G5 smartphone [84], Huawei smart watch [85] and JINS MEME glasses [86]. The smartphone was placed in the subject’s front left pocket of the jeans and it consists of 5 different sensory modalities: linear accelerometer (all sampled at 200 Hz), gyroscope, magnetometer (100 Hz), gravity, and 3-axis accelerometer. These sensory modalities are used to record body movement. Specifically, the linear accelerometer provides a three-dimensional sequence that specifies acceleration forces (excluding gravity) on the three axes. The gyroscope encodes three-dimensional angular velocities. The magnetometer sensor provides a three-dimensional sequence to describe the intensities of the earth’s magnetic field along the three axes which is quite useful to determine the smartphone’s orientation. The gravity sensing modality also generates a three-dimensional sequence that encodes the gravity forces on the three axes of the smartphone. The 3-axes accelerometer generates a three-dimensional sequence that specifies acceleration forces (including gravity) acting on the smartphone. Second, the smartwatch was placed on a subject’s left arm and it consists of two different sensory modalities: gyroscope and 3-axis accelerometer (both sampled at 100 Hz). Each of these sensing modalities generates a three-dimensional sequence of acceleration and angular velocities on the watch’s x, y, and z axes. These modalities are used to encode hand movements. Finally, the smart glasses are worn by the subject and it generates 3-dimensional data of accelerometer (sampled at 20 Hz). The accelerometer sensor in the smart glass provides three-dimensional acceleration information on the glasses’ x, y, and z axes which are used to record the head movement. Thus, the whole setup of activity encoding used eight sensor modalities through these wearable devices. The movement data of smart glasses and the watch is initially sent to the smartphone via Bluetooth connection and later all the recorded data sent to a home-gateway using a Wi-Fi connection. The entire process of recording is depicted in Figure 5.

The CogAge dataset contains 9700 instances of 61 different atomic activities obtained from 8 subjects. Among 8, there are 5 subjects who contributed to the collection of 7 composite activities too, using the aforementioned three wearable devices. There is one subject who only contributed to the collection of composite activities. Therefore, the dataset contains the composite activities of 6 subjects. An android application in a smartphone connects the smartwatch and glasses via Bluetooth, is used to record the composite activities. Thus, the whole recording setup provides a convenient and natural way for the subject such that he/she can move freely to the kitchen, washroom, or living room with these devices to perform daily life activities. More than 1000 instances of composite activities are collected, and missing data is removed during the pre-processing phase. Finally, the dataset comprises the 471 instances of left-hand activities (i.e., the activities are mainly performed using the left hand only) and 281 instances of both hands (i.e., the composite activities are performed using both hands). Therefore, the dataset contains in total of 752 instances of composite activities, and their description is outlined in Table 2.

The participants in data collection belong to different countries with diverse cultural backgrounds. Thus, their way of performing the same activity is also quite different (e.g., cooking). The versatility in performing the same activity makes the dataset much complex and a challenging task for the HAR systems to validate the generality of their methods, despite the low number of subjects. The data for composite activities was collected for training and testing phases separately in different time intervals. The length of every activity is not constant; it differs from 45 s to 5 min because some activities take a long time to be completed, like preparing food, and, on the other side, some activities take a shorter time, for example, handling medications. The atomic activity recognition algorithm [15] produced atomic scores after each time interval of approximately 2.5 s. We divided each composite activity into a window of size 45 s, i.e., 18 atomic scores vectors, for each composite activity instance. The longer instances were divided into multiple windows with a stride size of 6. The data of composite activities are divided into two parts: left-hand and right-hand activities data. Since the recording of each activity comprises a different number of instances, they are empirically reduced to a fixed number.

### 5.1. Experimental Setting

We performed three experiments each with three different experimental settings. In each experiment, the dataset is divided into training and testing sets differently. The experiments are performed using activities data which have been performed from left-hand (i.e., the smartwatch was placed on the subject’s left arm) and using both-hand (i.e., the smartwatch was either placed on a subject’s left or right arm), separately. The experimental settings are outlined in the following:

- *k*-fold cross-validation (CV): Training and testing data split on basis of *k* folds. We set the value of *k* = 3.
- Hold-out cross-validation: We used the data of 3 subjects for training and the data of the remaining 3 subjects for testing purposes, iteratively.
- Leave-one-out cross-validation: The data of 5 subjects are used for training, and the data of the remaining 1 subject is used for testing purposes, iteratively.

#### 5.1.1. Using Handcrafted Features

**Figure 6:**
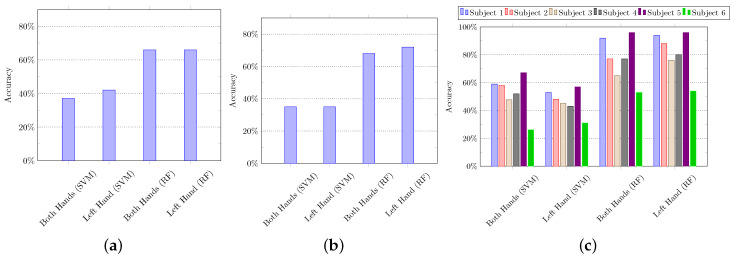
The composite activity recognition using handcrafted features with Support Vector Machine (SVM) and Random Forest (RF). (**a**) *K*-fold cross-validation, (**b**) hold-out cross-validation, and (**c**) leave-one-out cross-validation. In *k*-fold cross-validation, the training and testing data is split based on *k* folds. We set the value of *k* = 3. In hold-out cross-validation, the data of 3 subjects are used for training, and the data of the remaining 3 subjects is used for testing purposes, iteratively. In leave-one-out cross-validation, the data of 5 subjects are used for training, and the data of the remaining 1 subject is used for testing purposes, iteratively.

We computed 18 features on input data (i.e., atomic scores) as described in Section 4.2.1. These 18 features were computed against every column of features set of a single activity, i.e., 18 × total number of columns, and they are concatenated in a single row. Since the input data is 61-dimensional, the dimension of the handcrafted feature is 1×(18×61) (i.e., 1 × 1098). We used SVM and random forest (RF) to evaluate these computed features, and their recognition accuracies are summarized in Figure 6.

#### 5.1.2. Using Subspace Pooling Technique-Based Features

**Figure 7:**
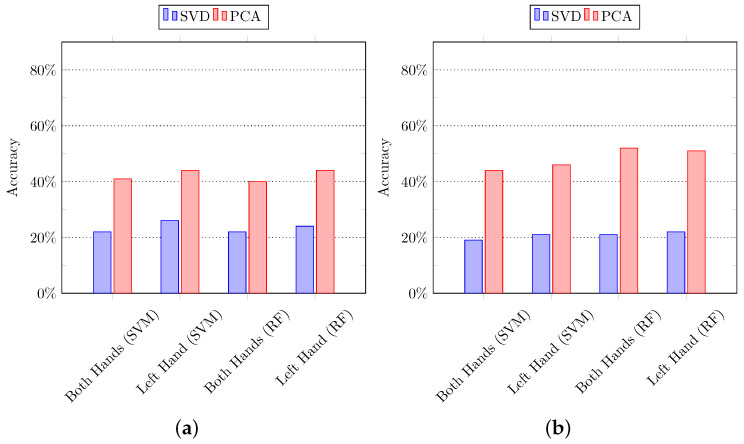
The composite activity recognition using features derived from the subspace pooling technique. The features are classified using Support Vector Machine (SVM) and Random Forest (RF). (**a**) *K*-fold cross-validation; (**b**) hold-out cross-validation.

**Figure 8:**
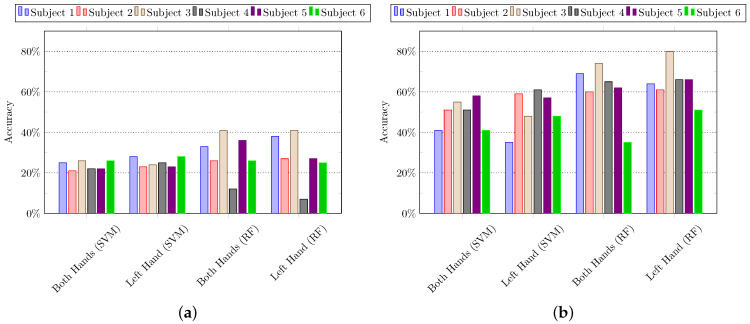
The composite activity recognition using features derived from the subspace pooling technique. The features are classified using Support Vector Machine (SVM) and Random Forest (RF). (**a**) Leave-one-out cross-validation: subspace pooling using singular value decomposition (SVD), and (**b**) leave-one-out cross-validation: subspace pooling using principal component analysis (PCA).

In the second set of experiments, the subspace pooling-based techniques are applied to the input data to project it into new dimensions to get the more robust representation of data in subspace [67]. In particular, we applied SVD and PCA on the input data, and their full-length features are used to recognize the composite activities using SVM and random forest algorithms. It is important to mention that all the features in new dimensions are used in the classification process. Figure 7 summarizes the results of experiments using k-fold cross-validation and hold-out cross-validation, whereas the results of leave-one-out cross-validation are summarized in Figure 8. It can be observed that the features extracted by using PCA performed quite well as compared to SVD.

#### 5.1.3. Using Optimal Feature Selection

We also assess the effectiveness of the optimal feature selection method on the data extracted using subspace pooling techniques. The features are selected based on the variance of eigenvectors (i.e., eigenvalues). Since there were 18 sequences in each activity of the original data and the dimension of each row is 61; they are concatenated in a 1-dimensional vector, i.e., 1×1098. The data of all the activities are arranged and the following steps are performed:

- First, we applied SVD and PCA separately and the matrix of eigenvectors (1098-dimensional) is extracted.
- Second, the sum of absolute values of every column of eigenvectors matrix is calculated, arranged in descending order with respect to its index number, and stored in a separate matrix.
- Third, original combined data was arranged with respect to the sorted sum of absolute eigenvectors.
- Fourth, the dimensions were reduced by iteratively selecting the small sets of features keeping in view the variance of eigenvectors.
- Finally, the selected set of features were evaluated using SVM and RF.

Similar to other experiments in the earlier categories, the *k*-fold cross-validation is first employed to split the data into training and testing sets, and the optimal features are classified using SVM and random forest. Table 3 shows the summarized results of composite activity recognition using *k*-fold cross-validation. In the second set of experiments, the hold-out cross-validation technique is employed, and the activities data of 3 subjects are used in the training set, whereas the rest activities data of 3 subjects are used in the testing set. Both training and testing sets are arranged according to the sorted absolute sum with their corresponding labels, and the selected features are classified using SVM and RF. Table 4 shows the summarized results of composite activity recognition using hold-out cross-validation. Similarly, the results of leave-one-out fold cross-validation are summarized in Table 5.

#### 5.1.4. Using HMM

In HMM classification, the objective is to calculate the probability of hidden states given the observation sequence. Hence, a sequence of observation and 7 models were constructed to find the model which best describes the observation sequence. In this experimental setting, the Gaussian HMM model with its hyperparameters, i.e., ‘n-components’, ‘covariance type’, ‘starting probabilities’, and ‘transmission matrix’, is used. The model is trained using two methods to compute the observation sequence, and the short detail of each method is described in the following.

**Figure 9:**
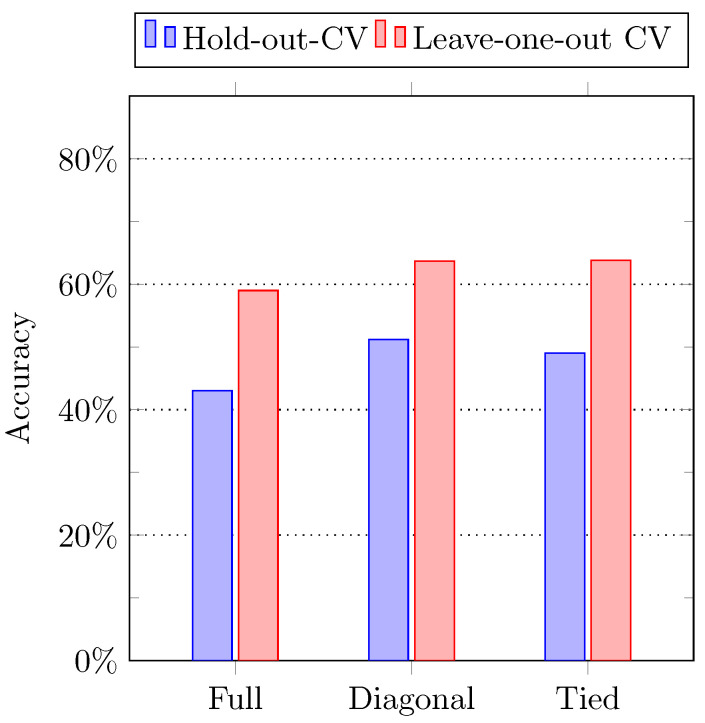
The composite activity recognition accuracies using Hidden Markov Model (HMM) classifier.

In the first method, the sequence of observation is calculated using the leave-one-out cross-validation technique. Since there are 6 subjects in the dataset, the Gaussian HMM model is trained using the activity data of 5 subjects, whereas the remaining 1 subject is used for testing purposes. We calculate the likelihood information while comparing the testing data to all the predicted observation sequences. The model with maximum likelihood was assigned to the input testing data. After getting all the models against all the testing data, the accuracy between the original models, and the estimated or predicted models is calculated. In the second method, the hold-out cross-validation technique is used to calculate the observation sequence. In particular, the activity data of 3 subjects is used in the model training, and the rest of the instances of 3 subjects are used for testing purposes. The maximum likelihood between trained and testing sequences is calculated as mentioned above. Figure 9 shows the result of the testing accuracy of both methods. It can be observed that the leave-one-out performed better than the hold-out cross-validation.

#### 5.1.5. Using Ensemble Classifier

Lastly, we also assess the performance of the ensemble classifier to recognize the activities. The idea is to obtain the prediction results from different classification algorithms, and the final result is computed based on the maximum voting technique. That is, human activities are recognized by combining all the predictions made by other individual classifiers. We employed 5 different classifiers with different feature representation of the same activity data: (1) SVM classifier using SVD-based features, (2) RF classifier using SVD-based features, (3) SVM classifier using PCA-based features, (4) RF classifier using PCA-based features, and (5) HMM model. All the models are trained with respective labels using the hold-out cross-validation technique to reduce the effect of overfitting or underfitting.

**Figure 10:**
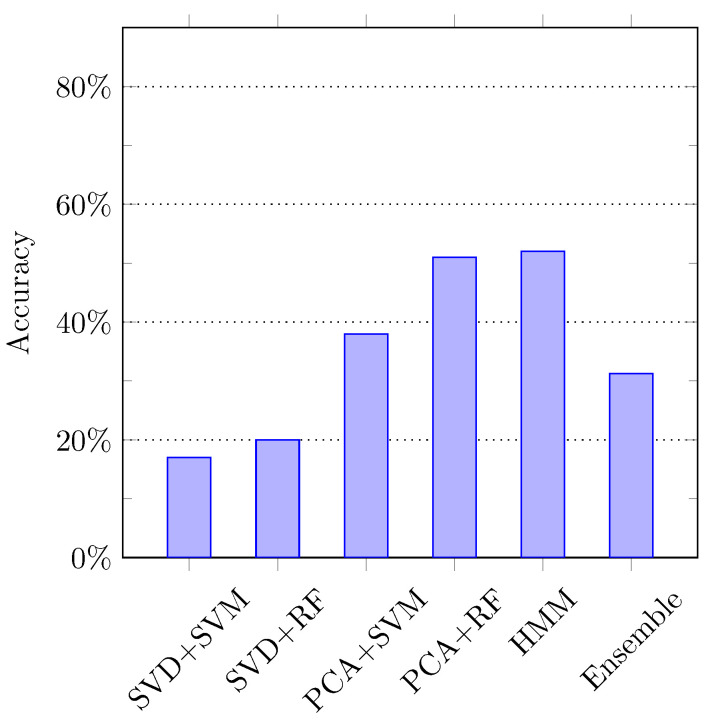
The composite activity recognition accuracies using ensemble classifier.

To obtain the SVD-based and PCA-based features, the same implementation is adopted as described in Section 4.2.2. The SVM classifier is trained with hyperparameter C=1, and the RF classifier is used with hyperparameter n=800, whereas the Gaussian HMM model is trained using hyperparameters *n*-component = 6, and covariance type = tied. The label information is gathered from each of the aforementioned 5 learning models and the label with maximum frequency is assigned to the respective instance of testing data. Figure 10 shows the comparison between the original results of individual classifiers and the ensemble classifier.

### 5.2. Discussion

This paper presents a technique to recognize the composite activities. We employed two different types of features for activity recognition: Handcrafted features and the features obtained using subspace pooling techniques. The experiments are carried out using three different settings: *k*-fold cross-validation, hold-out cross-validation, and leave-one-out cross-validation. A comparative analysis between all the techniques is presented in Table 6. It can be observed that handcrafted features perform quite well along with random forest classifiers to recognize the composite activities. Overall, it achieved average recognition accuracy of 79%.

The recognition results of the proposed features are also evaluated with state-of-the-art techniques [15]. A comparison analysis is carried out using two different experiments. Similar to Reference [15], in the first comparative analysis, the technique proposed in Reference [15] employed HMM and reported better recognition results using 3 states and 1000 iteration, whereas we also employed HMM by tuning the model hyperparameters, and the best results are achieved using 4 states, tied covariance matrix, and 1000 iterations. The recognition results in comparison with state-of-the-art techniques are summarized in Table 7. Second, the handcrafted features are used to recognize the composite activities using leave-one-out cross-validation. We performed 6 different experiments using handcrafted features, and all of them show good results. The results are shown in Table 8.

## 6. Conclusions

In this paper, a two-level hierarchical technique is proposed to recognize human activities using a set of wearable sensors. Since the human activity of daily life consists of several short sequences of actions (known as atomic activities), they are detected from the original sensory data and their recognition scores (in probabilities) are obtained. Later, the feature representation of composite activities is obtained from these atomic scores. We present two different methods for feature extraction: handcrafted and subspace pooling-based features. The proposed method is evaluated on a large public dataset and the recognition results of the composite activities are compared with existing state-of-the-art techniques which confirm its effectiveness. 

## Figures and Tables

**Table 1:** List of handcrafted features which are computed on the atomic scores.

Maximum	Skewness
Minimum	Kurtosis
Average	Auto-correlation
Standard-deviation	First-Order Mean (FOM)
Zero crossing	Norm of FOM
Percentile 20	Second-order mean (SOM)
Percentile 50	Norm of SOM
Percentile 80	Spectral energy
Interquartile	Spectral entropy

**Table 2:** Details of composite activities in the CogAge dataset [15]. The left hand represents the number of instances in which the activities are mainly performed using the left hand only, whereas, in the both hands setting, the activities are performed using both hands.

Total subjects:	6
Total activities:	7
Activities:	Brushing teeth
	Cleaning room
	Handling medications
	Preparing food
	Styling hair
	Using telephone
	Washing hands
Feature representation:	61-dimensional
Total number of instances:	752
Left hand instances:	471
Right hand instances:	281

**Table 3:** The composite activity recognition using a set of optimal features derived from the subspace pooling technique. The feature are classified with *k*-fold (where k=3) cross-validation. The average recognition accuracy with *k*-fold cross-validation is also presented.

	SVD		PCA	
	**All Hands**	**Left Hand**	**All Hands**	**Left Hand**
Accuracy (SVM)	47.04%	53.00%	47.04%	53.00%
Accuracy (RF)	46.00%	53.00%	46.00%	52.00%

**Table 4:** The composite activity recognition using a set of optimal features derived from the subspace pooling technique. The features are classified with hold-out cross-validation. The average recognition accuracy with hold-out cross-validation is also presented.

	SVD		PCA	
	**All Hands**	**Left Hand**	**All Hands**	**Left Hand**
Accuracy (SVM)	51.40%	54.01%	51.40%	54.67%
Accuracy (RF)	53.12%	55.99%	53.37%	55.99%

**Table 5:** The composite activity recognition using a set of optimal features derived from the subspace pooling technique. The features are classified with leave-one-out cross-validation. The average recognition accuracy with leave-one-out cross-validation is also presented.

	SVD		PCA	
	**All Hands**	**Left Hand**	**All Hands**	**Left Hand**
Accuracy (SVM)	64.93%	60.61%	58.31%	60.33%
Accuracy (RF)	61.53%	62.50%	62.05%	63.00%

**Table 6:** The comparative analysis of all the feature extraction techniques along with the classification algorithms. In all the experiments of leave-one-out cross-validation, the average recognition accuracies are reported here.

Handcrafted feature extraction	
Leave-one-out CV + SVM	48.9%
Leave-one-out CV + RF	79%
**Subspace pooling**	
PCA + Leave-one-out CV + RF	62.8%
**Optimal feature selection**	
SVD+ Leave-one-out CV + SVM	62.8%
**HMM**	
Leave-one-out CV	64%

**Table 7:** The comparison of the proposed method with existing state-of-the-art technique.

Method	Baseline	Proposed
Hidden Markov Model + Holdout CV	51.20% [15]	51.2%
Hidden Markov Model + Leave-one-out CV	61.01% [15]	64%

**Table 8:** The comparison of the proposed handcrafted features with different state-of-the-art techniques.

Proposed Handcrafted Features					
**SVM**			**RF**		
**k-Fold**	**Hold-Out**	**Leave-One-Out**	**k-Fold**	**Hold-Out**	**Leave-One-Out**
42%	35%	48.9%	66%	72%	79%
